# Intimate Partner Cyberstalking, Sexism, Pornography, and Sexting in Adolescents: New Challenges for Sex Education

**DOI:** 10.3390/ijerph18042181

**Published:** 2021-02-23

**Authors:** Yolanda Rodríguez-Castro, Rosana Martínez-Román, Patricia Alonso-Ruido, Alba Adá-Lameiras, María Victoria Carrera-Fernández

**Affiliations:** 1 Faculty of Education and Social Work, University of Vigo, 32004 Ourense, Spain; rosana.mr@uvigo.es (R.M.-R.); mavicarrera@uvigo.es (M.V.C.-F.); 2 Faculty of Education Sciences, University of Santiago de Compostela, 15782 Santiago de Compostela, Spain; patricia.alonso.ruido@usc.es; 3 Faculty of Social Sciences and Law, Carlos III of Madrid, 28903 Madrid, Spain; aada@emp.uc3m.es

**Keywords:** intimate partner cyberstalking, sexism, sexting, pornography consumption, adolescent, sexuality education

## Abstract

Background: Within the context of the widespread use of technologies by adolescents, the objectives of this study were to identify the perpetrators of intimate partner cyberstalking (IPCS) in adolescents; to analyze the relationship between IPCS and gender, age, sexting behaviors, pornography consumption, and ambivalent sexism; and to investigate the influence of the study variables as predictors of IPCS and determine their moderating role. Methods: Participants were 993 Spanish students of Secondary Education, 535 girls and 458 boys with mean age 15.75 (SD = 1.47). Of the total sample, 70.3% (*n* = 696) had or had had a partner. Results: Boys perform more sexting, consume more pornographic content, and have more hostile and benevolent sexist attitudes than girls. However, girls perpetrate more IPCS than boys. The results of the hierarchical multiple regression indicate that hostile sexism is a predictor of IPCS, as well as the combined effect of Gender × Pornography and Benevolent Sexism × Sexting. Conclusions: it is essential to implement sexual affective education programs in schools in which Information and Communication Technologies (ICT) are incorporated so that boys and girls can experience their relationships, both offline and online, in an egalitarian and violence-free way.

## 1. Introduction

The technological revolution has led to the increasing use of Information and Communication Technologies (ICT) by the adolescent population [1], thus establishing a new way of socializing through the virtual sphere [2]. In fact, some adolescents prefer online communication to face-to-face communication [3]. Thus, internet use, social media, and instant messaging are tools that boys and girls use routinely in both their peer and dating relationships [4,5]. Their growing impact on adolescents has become a major concern for educators and researchers in recent years [6]. As adolescents are at a crucial developmental stage in their lives in which new forms of interpersonal and affective relationships, such as falling in love, are experienced, new interests and needs emerge, as well as the first relationships, and also, the first sexual relationships [7].

Studies have identified the virtual sphere as a new space conveying many violent situations both in the peer group [8] and in dating relationships [9]. Thus, adolescents’ usage of ICT through online applications, video games, etc., should be considered useful to prevent violence and, specifically, partner violence [10]. Following the review carried out by Navarro-Pérez et al. [11] on ICT-based intervention tools, the following stand out for the prevention and intervention of Teen Dating Violence (TDV): Teen Choices program [12]; DetectAmor [13] and other mobile applications with a high level of effectiveness such as the Liad@s app [11,14], of an entertaining and educational nature, which aims to help adolescents to have egalitarian and non-toxic couple relationships, and involves having less sexist attitudes, identifying myths about love, and reducing situations of violence in their relationships. 

### 1.1. Intimate Partner Cyberstalking in Adolescents

Cyberstalking has its roots in traditional harassment or stalking. It is defined as a type of digital practice in which the aggressor exercises domination over the victim or victims through intrusion in their intimate life. This intrusion is repetitive, disruptive, and performed against the victim’s will [15]. This harassment includes false accusations, surveillance, threats, identity theft, insulting messages, etc., that generate fear in the victims [15]. The first episodes of cyberstalking occur between ages 12 and 17 years [16]. The conceptualization of intimate partner cyberstalking (IPCS) has a marked affective and/or sexual nature [15], as it is likely to be perpetrated against the partner or to be an approach strategy toward the ex-partner [17,18]. IPCS is considered a form of gender-based violence in young people, because it includes those behaviors that, through digital means, aim at domination, discrimination, and, ultimately, abuse of the position of power where the stalker has or has had some affective and/or sexual relationship with the harassed person [15]. Studies that have focused on IPCS in adolescents indicate that the most common behaviors are usually online control, online partner monitoring or online surveillance [19,20], concepts sometimes used interchangeably in diverse studies [21,22]. However, online control is a more serious behavior than online surveillance or online monitoring. Online surveillance or online monitoring is based on observing or carefully monitoring the partner or ex-partner to obtain information due to mistrust and insecurity [23], (e.g., “I get a lot of information about my partner’s activities and friendships from looking at his/her social media pages”), but control is to go one step further, because the purpose is to dominate and manage the life of the partner or ex-partner (e.g., “I have either asked my partner to remove or block certain people from their contacts [phone or social media], because I didn’t like the person, or I have done so myself [removed/blocked the person”]) [24]. The partner is often aware of the control they suffer by their boyfriend or girlfriend, unlike surveillance, which is more cautious [24,25]. Thus, international studies identify that between 42 and 49.9% of adolescents often check whether the partner is online on social media or instant messaging apps [26,27], between 19.5 and 48.8% of adolescents send constant or exaggerated messages to know where their partner is, what they are doing, or whom their partner is with [27,28], and between 32.6 and 45% of adolescents control who their partner is talking to and who they are friends with [26,28]. Qualitative studies also show that adolescents openly acknowledge that they often constantly check their partner’s mobile [25,29], that they share their passwords as a sign of commitment and trust, and that they often create fake profiles on social media to control their partners [19,30]. These online control behaviors show that adolescents consider them appropriate or acceptable, that is, these IPCS behaviors are normalized and adolescents even tend to justify them [19,25].

As for the prevalence rates of perpetration of IPCS in adolescents, international studies show great variability in the perpetrator. Early studies identified boys as the most frequent aggressors of IPCS [31,32]. However, the most recent studies indicate that IPCS aggressors are girls who tend more frequently to control and monitor their affective partners online [25,27,30]. In this sense, studies argue that boys tend to engage more in digital threatening and pressuring of their partner, especially when they want to have sex; whereas girls engage more in controlling behaviors to gain intimacy and exclusivity in their relationship [2,30] or even to preserve their relationship [31]. 

In Spain, the study of IPCS in adolescents is still an incipient line of research. The few existing investigations do not identify the IPCS perpetrator. There is great variability in the prevalence rates of IPCS; between 10% [33,34] and 83.5% [35,36] of adolescents admit that they control and monitor their partners online. In terms of frequency, according to the study of Donoso, Rubio, and Vilà [37], 27% of adolescents claim that they sometimes control their partner, and 14% sometimes inspect the partner’s mobile. In fact, 12.9% of adolescents ask their partner to text them to report where they are every minute [38]. In this sense, the study of Rodríguez-Castro et al. [4] shows that behaviors such as “controlling the time of the last connection” are common in adolescent partner relationships, without their identifying these behaviors as negative. Therefore, one of the objectives of this study is to evaluate the prevalence rate of IPCS, identifying the aggressor. 

### 1.2. Intimate Partner Cyberstalkxing in Adolescents

In order to further our knowledge of the IPCS phenomenon in adolescents, after reviewing the existing literature, other objectives of this study were to verify the relationship between IPCS and variables such as ambivalent sexism, sexting behaviors, and pornography consumption, as well as to predict which variables best explain IPCS. 

#### 1.2.1. Sexism and IPCS

We draw on the theory of Ambivalent Sexism [39], which describes ambivalent sexism as a two-dimensional construct made up of hostile and benevolent attitudes. Both sexisms function as complementary ideologies and as a reward and punishment system. Hostile sexism, with a negative tone, considers women inferior to men. Such hostile sexism is applied as a punishment to women who do not fulfill the traditional roles of wife, mother, and caregiver [40] In contrast, benevolent sexism, with a positive-affective tone, considers women to be different and, as such, it is necessary to care for and protect them, so traditional women are rewarded with benevolent sexism [41].

As international and national studies show, adolescents present ambivalent sexist attitudes, with boys having more hostile and benevolent sexist attitudes than girls [42,43]. In addition, the most sexist adolescents show more positive attitudes towards intimate partner violence [44]. In fact, studies show that both hostile sexism [45] and benevolent sexism [46,47] help explain intimate partner violence both in youth and adults [48,49].

In the online space, youth have found a new way to reproduce and perpetuate sexism [50]. Although we have found few studies that specifically link IPCS in adolescents to sexist attitudes, we can highlight the recent study of Cava et al. [33], which identified hostile sexism and relational violence as predictors of cyber-control strategies in boys, whereas myths of romantic love and verbal violence in the relationship were the main predictors of cyber-control in girls.

#### 1.2.2. Sexting and IPCS

The exchange of erotic/sexual and intimate content such as text messages, photos, and/or videos through social networks or other electronic resources—sexting—is a normalized reality in the relationships of adolescents both in and outside of Spain [4,27]. Thus, the figures point to a range of prevalence of sexting behaviors between 14.4 and 61% for adolescents, both in the international and national context [51,52]. 

Sexting behaviors are part of the strategies of intimate partner violence performed through sextortion [53]. Sextortion consists of blackmailing a person by means of an intimate image of themselves that they have shared over the Internet through sexting. The purpose of this blackmail is usually the domination of the victim’s will [53]. In fact, sexting behaviors due to the partner’s coercion have become one of the main reasons for youth’s participation in this behavior, especially girls [6]. Recent research points to the relationship between sexting practices in adolescents and intimate partner violence [54] but also, more specifically, cyber-control strategies in partner relationships [55], a trend reproduced in Spanish studies, which show how sexting practices in the couple are linked to the perpetration of cyberbullying [56,57]. Thus, girls who practice sexting with their partners are usually more likely to suffer some form of cyberbullying in their relationship [57].

#### 1.2.3. Consumption of Pornography and IPCS

Mainstream pornography has become a crucial social tool for the perpetuation of the patriarchal system because it helps shape women’s sexuality from the viewpoint of male self-interest. Through it, the patriarchal hierarchy is reproduced, confirming the attribution of a passive and silenced nature to women, and an active nature to men [58]. Through their free access to ICTs, our youth have become a consumer of pornographic content. International and national studies establish the prevalence of pornography consumption between 27 and 70.3% [59,60,61,62], with boys being more pornophile than girls [63,64]. The age range of initiation in pornography consumption is between 12 and 17 years [61,64], although some studies indicate that children are accessing pornography at increasingly younger ages, placing the first viewing at 8 years [60]. 

As Cobo [58] claims, the core of pornography intertwines masculine pleasure, domination, and violence. Adolescents acknowledge that pornography is violent, and 54% even admit to being influenced by it in their personal sexual experiences [61]. In fact, it has been found that boys who perform coercive behaviors and sexual abuse against their partner routinely view pornographic content [64]. However, we have not found any studies that directly relate pornography consumption to IPCS.

Taking into account this new context in which our young adolescents are socialized, the objective of this study was threefold: I. To identify IPCS perpetrators in the adolescent population; II. To analyze the relationship between IPCS and gender, age, sexting behaviors, pornography consumption, and ambivalent sexism; and III. To investigate the influence of the variables (gender, age, sexting behaviors, pornography consumption, and ambivalent sexism) as predictors of IPCS in the adolescent population.

## 2. Materials and Methods

### 2.1. Participants

Participants were 993 Spanish students of Secondary Education; 535 girls (53.9%) and 458 boys (46.1%). The age of participants ranged from 13 to 19 years, with a mean age of 15.75 years (SD = 1.47). One selection criterion of this study was to have a partner currently or to have had one in the past for at least six months. In this case, we found that of the total sample, 70.3% (*n* = 696) had a partner at the time of completion of the questionnaires or had had one in the past.

### 2.2. Instruments

An ad hoc questionnaire was used for this study. The questionnaire consisted of the following items and scales: 

#### 2.2.1. Demographic Questions 

The participants indicated their gender and age.

#### 2.2.2. Sexting Behavior

To identify sexting behaviors, we included the following question [65]: Have you ever sent sexually suggestive photos/videos or text messages of yourself? (1 = no, 2 = yes). 

#### 2.2.3. Pornography Consumption

To identify the consumption of pornography by adolescents, we included the following question: Have you ever searched for and/or viewed pornographic content over the internet? (1 = no, 2 = yes). 

#### 2.2.4. Inventory of Ambivalent Sexism in Adolescents (ISA)

The ISA [66] (based on the Scale of Ambivalent Sexism towards Women [40]) consists of 20 items that measure adolescents’ level of ambivalent sexism: 10 items measure hostile sexism and the remaining 10 items measure benevolent sexism. The response scale is a Likert-type scale ranging from 1 (strongly disagree) to 6 (strongly agree). Higher scores indicate higher levels of hostile and benevolent sexism. Cronbach’s alpha obtained in this study in the subscale of Hostile Sexism was 0.86, and in the Benevolent Sexism subscale, it was 0.85. 

#### 2.2.5. The Intimate Partner Cyberstalking Scale (IPCS-Scale)

This scale was developed “to measure specific behaviors of cyberstalking within an intimate relationship” (p.392) [24]. Examples of items include “I have checked my partner’s phone/computer history to see what they’ve been up to”, “I try to monitor my partner’s behaviors through social media”, and “I have used or have considered using phone apps to track my partner’s activities”. This scale consists of 21 items rated on a Likert-type response format ranging from 1 (strongly disagree) to 5 (strongly agree). Higher scores indicate greater engagement in IPCS behavior. The Cronbach alpha obtained in this study was 0.91. 

### 2.3. Procedures

Ethical approval was obtained from the PhD Program of the Education and Behavioral Sciences Ethics Committee prior to data collection. From a total of 20 public and secular Secondary Education centers of a province of northern Spain, we randomly selected 10 centers to participate in this study and, within each center, we selected the classrooms of the 2nd cycle of Compulsory Secondary Education and High School (Noncompulsory Secondary Education). The data collection process was carried out during the 2018/2019 school year. The questionnaires were applied in schools during regular school hours. The mean administration time was 25 min. Passive informed consent was received to administer the questionnaires, that is, the authorization of the academic community (directors and tutors).

### 2.4. Analysis

The following analyses were performed with the IBM SPSS v.21 (IBM Center, Madrid, Spain) program: first, the descriptive analyses: the mean (*M*) and standard deviation (*SD*) were calculated with Student’s *t*-test as a function of gender for the variables and scales studied. Cohen’s *d* was also used to evaluate the strength of the *f*^2^ effect size, whereby 0.02 is considered small, 0.15 is considered moderate, and 0.35 is considered large. Second, Pearson bivariate correlation coefficients (*r*) between the scales/subscales and the variables were calculated. Third, Hierarchical Linear Regression was used to test the regression model and interaction effects. The predictor variable was IPCS. The variables gender, age, sexting behavior, and consumption of pornography were entered in Step 1 of the regression model; next, hostile sexism and benevolent sexism were entered in Step 2. Interaction terms (Predictor x Predictor) were entered in Step 3 of the model to test the interactions between combinations of variables of the study. Beta coefficients (β) and Student’s *t*-test indicated the proportion of the unique effect contributed by each predictor variable. The coefficient of determination (*R*^2^), adjusted coefficient (Δ*R*^2^), ANOVA (*F*), and *p*-values were used to examine significant effects in the regression model.

## 3. Results

First, we compared the differences in the means of IPCS, sexting behavior, consumption of pornography, and hostile and benevolent sexism as a function of gender. As can be observed in Table 1, there were significant differences in all the scales/subscales, with a variable effect size. Boys carried out the most sexting behaviors (t = 8.07, *p* < 0.001, *d* = 0.61), consumed more pornographic content (t = 11.19, *p* < 0.001, *d* = 0.84), were more hostile sexists (t = 6.89, *p* < 0.001, d = 0.52), and were also more benevolent sexists (t = 3.97, *p* < 0.001, *d* = 0.30) than their female classmates. However, girls perpetrated more IPCS than boys. 

All the bivariate correlations between the scales and subscales of the study (see Table 2) were significant. Gender was found to be positively related to IPCS (r = 0.10, *p* < 0.01) and negatively to hostile sexism (r = −0.2510, *p* < 0.001), benevolent sexism (r = −0.15, *p* < 0.001), sexting behaviors (r = −0.29, *p* < 0.001), and pornography consumption (r = −0.38, *p* < 0.001). That is, girls carried out more cyberstalking behaviors towards their partners, whereas boys were the most hostile and benevolent sexists who performed the most sexting and consumed more pornographic content.

It was also found that IPCS correlated positively with hostile sexism (r = 0.32, *p* < 0.01), benevolent sexism (r = 0.39, *p* < 0.01), sexting behaviors (r = 0.32, *p* < 0.01), and pornography consumption (r = 0.33, *p* < 0.01). That is, people with high IPCS had a higher level of hostile and benevolent sexism, practiced more sexting, and consumed more pornographic content.

In addition, sexting behaviors and pornography consumption correlated positively with age (r = 0.10, *p* < 0.01; r = 0.11, *p* < 0.01), hostile sexism (r = 0.33, *p* < 0.01; r = 0.36, *p* < 0.01), benevolent sexism (r = 0.32, *p* < 0.01; r = 0.34, *p* < 0.01), and IPCS (r = 0.32, *p* < 0.01; r = 0.33, *p* < 0.01) whereas they correlated negatively with gender (r = −0.29, *p* < 0.001; r = −0.38, *p* < 0.001). That is, the people who performed more sexting and consumed more pornography were older, the most sexist (hostile and benevolent), and performed the most cyberstalking of their partner; also, boys practiced more sexting and consumed more pornography. A positive and strong correlation was also obtained between sexting and pornography consumption (r = 0.64, *p* < 0.01), so those who viewed more pornographic content were also more active in sexting behaviors.

Next, the regression model was tested using hierarchical multiple regression to compare the strength of the prediction estimates of the variables (participants’ gender, age, sexting, and pornography consumption) for IPCS (see Table 3). The three variables were entered at Step 1 of the analysis, accounting for a significant 20.3% of the variance in IPCS. 

At Step 2, the two predictor variables (hostile and benevolent sexism) were entered in the regression analysis, which accounted for a total of 29.5% of the variance in the model as a whole. The addition of the predictor variables accounted for an additional 9.2% of the variance in IPCS, ΔR^2^ = 0.092, F(2, 674) = 46.90, *p* < 0.001. In the final model, hostile sexism (β = 0.12, t = 2.83, *p* = 0.01)) was significant. 

Two-way interaction terms between Gender × Pornography Consumption and Benevolent Sexism × Sexting, were entered independently in Step 3 of the model using an interaction variable (Predictor × Predictor). Two predictors in the combined effect of Gender × Pornography Consumption (β = 0.34, t = 2.01, *p* = 0.001) and Benevolent Sexism × Sexting (β = 0.15, t = 1.69, *p* = 0.01) were significant. All other combinations of interactions were non-significant.

**Figure 1:**
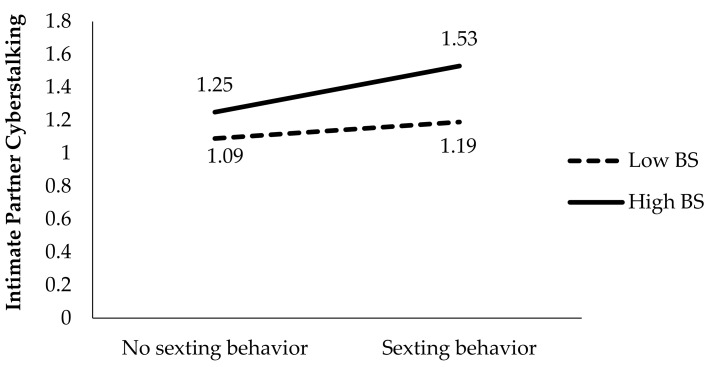
Moderating effect of benevolent sexism (BS) between sexting behavior and intimate partner cyberstalking.

**Figure 2:**
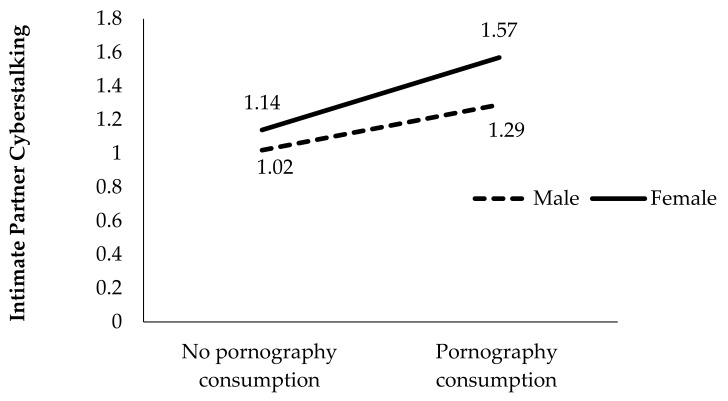
Moderating effect of gender on pornography consumption and intimate partner cyberstalking.

To clarify the meaning of these two significant interactions of the hierarchical regression, a detailed analysis of the mean scores in the IPCS scale obtained by each of the groups in each of the interactions was carried out. These mean scores for each group are presented in Figure 1 and Figure 2.

As shown in Figure 1, we compared the mean scores in pairs with a *t*-test. These comparisons indicated that students with a high level of benevolent sexism carried out more IPCS behaviors than those with a low level of benevolent sexism, both among those who did not practice sexting (t = −3.45, *p* < 0.001) and those who did practice sexting (t = −6.29, *p* < 0.001). Likewise, students who practiced sexting scored higher in IPCS than those who did not practice it, both among those with high benevolent sexism (t = −4.92, *p* < 0.001) and those with low benevolent sexism (t = −2.56, *p* < 0.001). Therefore, the benevolent sexist students who carried out sexting behaviors scored higher in IPCS than all the other groups (that did not practice sexting). Therefore, the results indicate that the relationship between sexting practices and the perpetration of IPCS was moderated by the level of benevolent sexism.

Similarly, we compared the mean scores using *t*-tests in Figure 2. We note that girls obtained higher scores for IPCS than boys, both among those who did not consume pornographic content (t = −7.32, *p* < 0.001) and those who did consume it (t = −5.77, *p* < 0.001). In addition, students who consumed pornographic content, whether they were boys (t = −9.70, *p* < 0.001) or girls (t = −9.80, *p* < 0.001), performed more IPCS behaviors than those who did not consume pornography. Moreover, girls who consumed pornographic content scored higher than all the other groups in IPCS. Therefore, the results indicate that the significant relationship between pornography consumption and IPCS was moderated by gender.

## 4. Discussion

Numerous studies have shown the influence of isolated variables such as gender [24], personality traits [18], sexism [67,68], beliefs about love [68], sexting [57], or the consumption of pornography [69] on violence or cyber-violence in couple relationships, although mainly in the adult population and university students. To our knowledge, no study has combined the variables of this study and clarified their moderating effect on adolescents regarding IPCS. 

Initially, this study analyzed the prevalence of IPCS in adolescents based on gender. Although low means were obtained in IPCS, adolescent girls claimed to perform more cyberbullying behaviors towards their partners and also stated that they would reproduce these online harassment behaviors if they had any kind of suspicions about their partner. These results are in line with international [27,30] and national [4,57] studies that show that girls perform more cyber-control of their partners. These results show a turning point in the profile of the cyber-control aggressor in couples when compared to traditional gender-based violence in adolescence when boys were the main aggressors [31,70]. Now, the girls aggress more than the boys.

Other interesting results of this study in line with international and national studies is that boys carry out more sexting behaviors than girls [63,65,71] and they also consume more pornographic content compared to girls [60,64]. We also found that older boys and girls practice the most sexting [65] and consume more pornographic content over the internet [60,61]. As our results show, pornography consumption and sexting are strongly related, such that the more pornographic content boys and girls consume, the more sexting behaviors they perform. Although few studies explored this association, the study of Stanley et al. [64], involving adolescents from five European countries, also demonstrates this strong linkage. The research of Romito and Beltramini [72] went so far as to conceptualize sexting as a means through which adolescents produced their own pornographic content that they later sent to others. 

Our results show that adolescents continue to present sexist attitudes. Boys also have higher levels of ambivalent sexism (hostile and benevolent) than girls. However, the greatest differences concern hostile sexism. These results are coincident with numerous studies [42,47]. It is also interesting to note that, despite differences as a function of gender, both boys and girls increased their level of more subtle sexism (benevolent), which, due to its positive-affective tone, masks situations of discrimination against women, causing many young people to be unable to identify it. We also found that both hostile and benevolent sexism were positively related to pornography consumption and sexting behavior. Hence, boys and girls with more sexist attitudes consumed the most pornographic content and performed more sexting behaviors.

When we examined the relationship between IPCS and sexting behaviors, pornography consumption, and ambivalent sexism, we found that IPCS was positively related to every one of them. Thus, the boys and girls who exercised more cyber-control of their partners were more sexist (hostile and benevolent), performed more sexting behaviors, and also consumed more pornographic content. Various studies consider sexism, especially hostile sexism, as a predictor of violence or cyber-violence in the couple [33,73]. International literature also links sexting practices to cyberstalking in couples [6], but this is the first study to relate all these variables. 

Finally, our focus was on determining the influence of gender, age, sexting behavior, pornography consumption, and ambivalent sexism as predictors of IPCS as well as confirming their moderating role in adolescents. This is the first study that examines the combination of these variables. The results obtained identified hostile sexism and interactions combining the effect of gender and pornography consumption and the effect of benevolent sexism with sexting as predictors of IPCS. It is again confirmed that the level of hostile sexism has become a key variable that predicts online control of the partner. Therefore, the most hostile sexist adolescents are more likely to perform IPCS behaviors. In this case, gender and the level of benevolent sexism modulate cyberstalking behavior in the couple. Therefore, our results show that girls who consumed more pornographic content cyberstalked their partner more. In addition, more benevolent sexist boys and girls who performed more sexting behaviors tended to cyber-monitor their partner more. 

These results encourage us to take a step further and reflect on why the more benevolent sexist adolescents perform more sexting and also cyber-monitor their partners more, and why girls—greater pornography consumers—engage in more cyberstalking in their relationships than boys. It is clear that the digital scenario has become a new space to perpetrate violence through online control and surveillance of the partner [2]. Although both boys and girls admitted to controlling their partner in the virtual space, we found that girls cyber-monitored their partner more and also consumed more pornographic content. At the same time, male and female adolescents with ambivalent attitudes (hostile and benevolent)—with boys being more sexist and performing more sexting [65]—cyber-monitor their partner. 

Given these results, the most plausible explanation lies in the differential socialization. Both boys and girls are educated based on gender stereotypes [74]. Thus, boys are educated as an “autonomous self”, stressing independence, power, and oriented toward competitiveness. Girls are educated in the ethics of care, emotionality, and dependence, and they build their identity based on an “I in relation” to others, on commitment to the partner, granting love a central place in their life [75,76]. This makes girls yearn to have a partner because it gives them a sense of security and a position, social recognition, and protection within the peer group [77]. Thus, adolescent girls clearly recognize the value of “being someone’s girlfriend” and are afraid of losing “the girlfriend status” in the peer group [77] (p. 208). This shows that relationships are still conditioned by patriarchy and a conception of androcentric sexuality that implies that girls "without a partner" can be attacked, rejected, or ignored by the peer group [77]. On the one hand, the fear of losing their partner possibly pushes girls to become consumers of pornographic content, in order to reproduce their total dedication to the male’s desire in their sexual practices. On the other hand, emotional dependence on their partner, coupled with jealousy and mistrust, causes violence to materialize through their cyber-control [4,19,30,53]. In fact, both boys and girls consider cyber-control as harmless, not a form of violence, and they may even regard it as play [25]. Thus, they see controlling behavior as a way to express love, care, and affection toward a partner and also as an “effective” tool to maintain their couple relationship [24,31]. Therefore, it is necessary to provide our youngsters with the necessary tools to demystify these cyber-behaviors that they have normalized in their relationships. 

The main limitation of this study is related to the sample, which consisted of Secondary Education students from public and lay educational centers, discarding students of the same educational level who were enrolled in private and religious schools. It would also be interesting to incorporate new variables related to the possession and use of technologies and also to include scales of cyber-violence in the couple that can specifically detect certain behaviors such as control, online jealousy, and threats, among others. In the future, further deepening the study of intimate partner cyberstalking in the adolescent population should be addressed from a qualitative perspective in which boys and girls discuss in their own words their beliefs, attitudes, and behaviors about cyberstalking in their relationships. 

## 5. Conclusions

In relation to the results obtained with adolescents who present sexist attitudes, consume pornography, practice sexting, and carry out behaviors of cyber-monitoring of the partner—highlighting girls’ increased participation in this type of violence—, we are faced with the need to train adolescents in the field of affective-sexual education. In Spain, the current Organic Law for the Improvement of Educational Quality [78] formally maintains the value of freedom and tolerance to promote respect and equality, although, at a practical level, it was a setback because it eliminated the academic subjects to address the contents of sex education [79]. 

In Spain, the most widespread sex education model is anchored in a moral/conservative model that demonizes sexuality and a risk/prevention model that uses fear and disease as keys to learning. Both these models reproduce the traditional, sexist, and heteronormative view of affective-sexual relationships [80]. The purpose of sex education should be to create a model of liberating, critical, and emancipating sexuality; for this purpose, it is necessary to have adequate comprehensive sexual training [81].

As the results of this study show, we cannot forget that the context in which young people currently live has changed drastically [82]. Thus, with the incorporation of ICTs—the Internet, social networks, etc.—on the one hand, a space is opened up to new opportunities for the promotion of sexual and reproductive health, but, on the other hand, new phenomena also arise (such as sexting, cyber-monitoring, etc.) that can make adolescents vulnerable [25,65]. Therefore, ICTs, which have encouraged dispersion of information, have become opinion-makers of the youngest population [83], and a powerful transmitter of messages, many of them erroneous or biased, about sexuality, and focused specifically on how sexual relations between men and women should be [79]. Pornography is the main vehicle for transmitting a conceptualization of androcentric and violent sexuality for younger people [58]. The increasing impact of its consumption influences their relationships, introducing certain levels of violence into sexual practices and consolidating the patriarchal imaginary of inequality between men and women [60], placing male pleasure at the center, and relegating female pleasure [58].

In short, it is essential to implement sexual education programs in schools incorporating ICTs for their safe and responsible use [84]. Several studies have tested the high effectiveness of teaching tools in version 4.0 (audiovisual materials, telephone apps, etc.) focused on the prevention of gender-based violence, which are at the service of the educational community (educators, mothers/fathers, and students) [10], such as the Liad@s mobile app to work from a playful perspective such important aspects as ambivalent sexism (hostile and benevolent), myths about love, and egalitarian relationships [10,11]. Sex education programs should be integrated into the curriculum at all levels of education as just one more subject [79], addressing essential content such as: body identity, gender identity (sexism, gender stereotypes, sexual orientation, etc.), self-esteem and self-concept, emotions, egalitarian socio-affective relationships (love, infatuation, friendship, etc.), sexual behavior, and sexual health [85] and relying on the various ICT tools of that combine learning, motivation, and fun [14]. Only in this way will the current educational system be able to respond to these new social realities generated both online and offline to allow boys and girls to live and express their interpersonal and couple relationships in an equal and violence-free way.

## Figures and Tables

**Table 1:** Differences in the means of scales/subscales by gender.

**Intimate Partner Cyberstalking**	**Mean (SD)**		**t**	***p***	***d*-Cohen**
	**Female**	**Male**			
	1.31 (0.41)	1.23 (0.39)	−2.69	0.007	−0.20
Sexting behavior	1.40 (0.49)	1.69 (0.46)	8.07	0.001	0.61
Pornography consumption	1.40 (0.49)	1.78 (0.41)	11.19	0.001	0.84
ISA_Hostile sexism	1.66 (0.74)	2.12 (0.96)	6.89	0.001	0.52
ISA_Benevolent Sexism	2.11 (0.97)	2.42 (1.08)	3.97	0.001	0.30

Note: SD: standard deviation; t: Student’s *t*-test; *p*: level of signification; *d*-Cohen: Cohen’s d effect size

**Table 2:** Pearson correlations between the various scales/subscales.

Scales	Gender	Age	2	3	4	5	6
(2) Intimate Partner Cyberstalking	0.10 **	0.07	-				
(3) ISA_Hostile Sexism	−0.25 ***	0.03	0.32 **	-			
(4) ISA_Benevolent Sexism	−0.15 ***	0.04	0.39 **	0.58 **	-		
(5) Sexting behavior	−0.29 ***	0.10 **	0.32 **	0.33 **	0.32 **	-	
(6) Pornography Consumption	−0.38 ***	0.11 **	0.33 **	0.36 **	0.34 **	0.64 **	-

Note: ** *p* < 0.01; *** *p* < *0*.001. Gender (1 = boys; 2 = girls).

**Table 3:** Hierarchical linear regression analysis predicting intimate partner cyberstalking.

Predictors	Step 1		Step 2		Step 3	
	β	*t (p)*	β	*t (p)*	β	*t (p)*
Gender	0.28	7.62 ***	0.30	8.52 ***	−0.17	−1.26
Age	0.01	0.70	0.01	0.10	0.01	−0.198
Sexting Behavior	0.20	4.58 ***	0.14	3.34 ***	−0.43	−1.43
Pornography Consumption	0.31	6.74 ***	0.23	5.20 ***	−0.24	−1.20
ISA_Hostile Sexism			0.12	2.83 **	0.11	2.65 **
ISA_Benevolent Sexism			0.25	6.15 ***	−0.68	−0.491
Gender × Pornography Consumption					0.34	2.01 ***
Benevolent Sexism × Sexting Behavior					0.15	1.69 **
*F* (df, df _error_)	42.98 (4, 676) ***		46.90 (2, 674) ***		24.39 (7, 667) ***	
*R* ^2^	0.203		0.295		0.322	
Δ*R*2	0.203		0.092		0.028	
ΔF2	42.98 ***		43.83 ***		3.89 ***	

Note: ** *p* < 0.01; *** *p* < *0*.001. Gender (1 = boys; 2 = girls). **β**: Beta coefficients. R^2^: coefficient of determination. ΔR^2^: adjusted coefficient. F: ANOVA. t: Student’s *t*-test and *p*-values.

## Data Availability

The data presented in this study are available from the corresponding author on reasonable request.
